# Parenting styles and freshman’s internet gaming disorder: a moderated chain mediation model involving presence of meaning in life and self-control

**DOI:** 10.3389/fpsyg.2026.1748294

**Published:** 2026-04-13

**Authors:** Hanyue Jia, Yaozhong Zhang, Azeem Obaid, Fatma Sulaiman, Haidong Zhu

**Affiliations:** 1 Normal College of Shihezi University, Xinjiang, China; 2 Psychological Application Research Center, Shihezi University, Shihezi, China

**Keywords:** freshman, internet gaming disorder, parenting styles, presence of meaning in life, self-control

## Abstract

**Objectives:**

This study aimed to examine the impact of parental rearing styles on internet gaming disorder among college students, with a particular focus on the mediating roles of presence of meaning in life and self-control through the mediating pathway as well as the moderating effect of gender.

**Methods:**

Data were collected from 728 Chinese first-year undergraduates in Western China in 2025, using self-report measures that assessed parenting styles, presence of meaning in life, self-control, and internet gaming disorder. The cross-sectional survey data were analyzed using SPSS 27.0 and PROCESS *Macro* 4.1, bootstrapping with 5,000 resamples to test for mediation and moderated mediation effects.

**Results:**

Negative parenting styles, such as rejection and over protection, were positively associated with internet gaming disorder, both directly and indirectly through the chain mediation of presence of meaning in life and self-control. In contrast, positive parenting style such as emotional warmth did not directly predict internet gaming disorder but exerted a fully indirect effect via the same chain pathway. Indeed, gender moderated the relationship between presence of meaning in life and self-control, with females showing stronger mediation effects. Under positive parenting style, females exhibited greater protective effects against internet gaming disorder; under negative parenting styles, they were more vulnerable to internet gaming disorder though the chained mediation effects.

**Conclusion:**

Interventions should highlight the importance of improving positive parenting styles and reducing negative ones. Efforts should focus on cultivating the presence of meaning in life and strengthening self-control. Particular attention should be given to females raised under negative parenting styles, with early identification of their risk for Internet gaming disorder and the provision of timely psychological support. These strategies are essential for effectively addressing Internet gaming disorder among college students.

## 1 Introduction

In the digital age, the widespread use of the internet and smartphones has accelerated the rapid global expansion of online gaming. Data indicate that the number of gamers worldwide has surpassed 2.8 billion, with the Asia-Pacific region holding a dominant position (Lidia Research and Pocket Gamer, 2025). Concurrently, China’s mobile gaming user base has reached 663 million, and e-sports users have grown to 490 million (He et al., 2024; CADPA and GameLook, 2024).

As a prevalent form of entertainment, online gaming has dual implications: moderate online gaming can satisfy individuals’ entertainment needs and promote the development of cognitive abilities (Granic et al., 2014; Green and Bavelier, 2003). However, excessive gaming leading to Internet Gaming Disorder (IGD)—classified as a mental disorder in DSM-V—has been associated with various negative consequences on psychological, social, and physical health that extend from adolescence into adulthood (Lin et al., 2021; Sugaya et al., 2019).

Previous review studies have indicated that the prevalence of Internet Gaming Disorder (IGD) among emerging adults is higher than that observed in other age groups (Gisbert-Pérez et al., 2026). From a developmental psychological perspective, emerging adulthood is characterized by the coexistence of identity exploration, increasing autonomy, and heightened susceptibility to risk behaviors (Arnett, 2000). The transition into university represents a critical period marked by a shift from external regulation to self-regulation, making the beginning of college a particularly important window for examining behavioral risk mechanisms. Empirical findings further support this developmental argument. Compared with adolescents, first-year college students exhibit a higher risk of IGD (Gao et al., 2022), and problematic internet use has been shown to peak during the freshman year. This heightened vulnerability stems from the interaction between their developmental characteristics and challenges in adapting to university life.

On one hand, as “digital natives,” they exhibit traits such as dependence on digital technology, preference for multitasking, reliance on visual communication, and a strong desire for instant gratification (Teo, 2013). On the other hand, as “emerging adults,” their prefrontal cortex remains underdeveloped, leading to weaker impulse control and greater susceptibility to gaming temptations (Teng et al., 2020).

Moreover, the widespread use of smartphones has blurred the boundaries between learning and entertainment, making young adults more prone to excessive digital device use (Sohn et al., 2019). Regarding adaptation challenges, the transition from high school to college imposes multiple pressures—social, environmental, and academic —that make freshmen more likely to perceive online gaming as a means of “relaxation and escapism” (MIDiA Research, 2025). This transitional period thus represents a critical window for intervention. Therefore, exploring the causes of IGD among freshmen holds significant practical importance for promoting their psychological development.

Although university life marks a new chapter of independence, parental influence remains profound (Cheng et al., 2023). According to ecological systems theory, the family as a microsystem exerts direct, stable, and cross-contextual effects (Schoeps et al., 2020; Sang and Xi, 2005), significantly impacting the healthy development of adolescents’ psychological and social competencies (Yang and Song, 2025). The I-PACE model proposed by Brand et al. (2019) provides a theoretical framework for understanding the pathways through which parenting styles affect gaming addiction among freshmen. Based on the I-PACE model (Li et al., 2023) (Interaction of Person-Affect-Cognition-Execution), when individuals (such as freshmen) experience maladaptive parenting styles, they may view online gaming as a compensatory pathway to fulfill their psychological needs (Laconi et al., 2017). The resulting temporary satisfaction encourages individuals to form new cognitive patterns, leading them to repeat internet gaming at higher frequencies. This further perpetuates a negative compensatory cycle, ultimately escalating to IGD. Previous studies have shown that negative parenting styles—such as authoritarian parenting (Zhang et al., 2017) and parental perfectionist expectations (Meng et al., 2025)—can contribute to this addiction.

However, although the I-PACE model and related perspectives provide a useful theoretical basis, several important gaps remain in the existing literature.

Despite growing research on parenting styles andIGD, several important gaps remain.

First, from a theoretical perspective, compensatory Internet use theory emphasizes that individuals engage in online activities to compensate for offline psychological deficits. However, how parenting styles shape such compensatory motivations in the context of gaming remains underexplored. In particular, limited research has integrated meaning-related cognitive resources (e.g., presence of meaning in life) into the compensatory framework to explain how family environments translate into gaming-related addictive behaviors.

Second, most empirical studies on IGD have predominantly focused on adolescents. Although emerging adults and college students represent a population undergoing significant developmental transitions and autonomy expansion, relatively fewer studies have examined mechanistic pathways linking parenting styles to IGD in this group. Moreover, recent integrative evidence in higher education contexts has highlighted the psychological impact of helicopter parenting and overinvolvement, yet its connection to gaming-related addictive outcomes—especially through cognitive and executive mechanisms—remains insufficiently theorized.

Third, although meaning in life and self-control have increasingly been incorporated into IGD models, existing studies often treat gender merely as a control variable. Few studies have examined whether gender moderates the sequential transformation of cognitive resources into executive regulation within a theoretically grounded mediation chain.

Accordingly, the present study integrates parenting styles, presence of meaning in life, and self-control within the I-PACE framework, and examines gender as a moderator of the cognitive-to-executive transformation process. By adopting a process-oriented perspective, this study aims to clarify how and under what conditions family environments contribute to IGD risk among college freshmen.

### 1.1 The relationship between parenting styles and IGD

Darling and Steinberg (1993) synthesized prior research to redefine parenting styles as the integration of parental educational philosophies, behaviors, and emotional expressions. This perspective is characterized by stability and profound significance for individuals’ development.

First, parenting styles exert both immediate and long-term influences on shaping individual behavior. Bronfenbrenner’s Ecological Systems Theory posits that individual behavior does not arise in isolation but is shaped by ongoing interactions between the individual and environmental systems, with the family playing a pivotal role in the socialization process (Bronfenbrenner, 1979). Xie and Chen (2021), analyzing cross-sectional and longitudinal data from university freshmen, concluded that parental parenting styles continue to influence children’s behavior even after they enter early adulthood. These styles indirectly affect maladaptive behaviors among first-year college students upon enrollment and subsequently, further revealing the cross-contextual and stable nature of parenting styles.

Second, different parenting styles exert varying effects on college students’ IGD. Existing research indicates that parents employing positive parenting styles can reduce online addiction among college students while fostering higher relationship quality (Anasuyari and Latifah, 2023). Conversely, adolescents experiencing negative parenting or family conflicts are more likely to develop IGD (Petrescu et al., 2025). This further supports the notion that individuals may immerse themselves in virtual environments to buffer adverse psychological effects of negative parenting. In summary, this study proposes Hypothesis 1: Positive parenting styles inhibit IGD, while negative parenting styles promote it.

### 1.2 Mediating role of presence of meaning in life

The transition to university life indicates a critical period for the development of meaning in life (Steger et al., 2009). Frankl (1966) theory posits that individuals who lose their sense of life’s meaning fall into an “existential vacuum,” manifesting as emptiness, aimlessness, and value confusion. Those lacking life meaning may seek validation through virtual achievements. This theory has been operationalized in psychology as “meaning in life,” defined as “the understanding of one’s own existence and the perception of its significance” (Steger et al., 2006). Conceptually, Steger et al. (2006) further divided meaning in life into two dimensions based on Frankl (1966) theory: the presence of meaning (PML) and the search for meaning (SML). PML represents the cognitive dimension, reflecting the extent to which individuals comprehend their lives, perceive purpose, and feel that their existence is meaningful. SML constitutes a motivational dimension, involving active efforts to establish or strengthen one’s understanding of life’s meaning.

Notably, PML exhibits a significant positive association with positive psychological indicators and a significant negative correlation with negative psychological indicators (Kleiman et al., 2013; Park et al., 2010). It serves as a critical protective factor for mental health, buffering against issues like depression and anxiety (Cohen and Cairns, 2012). For instance, Park et al. (2010) found that individuals with a stronger PML experienced reduced depressive symptoms associated with the search for meaning. In contrast, the relationship between SML and mental health is more complex and debated, with studies linking it to both positive (Bailey and Phillips, 2015) and negative outcomes (Li et al., 2019; Steger et al., 2008).

Overall, PML is a more stable and robust predictor of mental health than SML, demonstrating stronger correlations, greater predictive power, and broader relevance across psychological traits. Its protective effect is particularly pronounced among individuals with traumatic experiences or lower baseline mental health (Doran et al., 2014), and its association with mental health shows good temporal stability (Steger et al., 2008).

Within this context, existential psychology provides a valuable theoretical framework for understanding the mechanisms linking parenting styles to IGD among college students. Positive styles characterized by warmth and understanding enhance one’s overall meaning in life, whereas negative styles involving overprotection, rejection, and emotional neglect diminish it (Zhu et al., 2011; Li et al., 2021; He et al., 2023). For example, a survey of 542 college students by Liu et al. (2022) found significant negative correlations between parental rejection/overprotection and meaning in life, and significant positive correlations between parental emotional warmth and meaning in life.

Research on adolescent internet gaming disorder consistently highlights the prominent role of PML, the core cognitive dimension of meaning in life, in reducing addictive behaviors across different populations. Focusing on left-behind middle school students, Wang et al. (2022) found that while SML was unrelated to pathological internet use, PML showed a significant negative correlation. This perception could independently or in conjunction with self-stigma form a chained mediating pathway, buffering the positive influence of sensitivity rejection on pathological internet use. This indicates that the clearer the actual perception of life’s meaning, the lower the risk of internet addiction among left-behind middle school students.

Among university students, studies on university students—by Cao Ruilin et al. in Northeastern China (Cao et al., 2019) and Jilin Province (Cao et al., 2023), and by Zhao et al. (2020a,b) on female students’ mobile phone addiction—have consistently validated that higher levels of PML are associated with a lower likelihood of internet or mobile phone addiction, either directly or indirectly through mediators like self-esteem and school adaptation. “Presence of meaning in life” consistently emerges as a crucial protective factor in reducing addiction risks.

This body of evidence indicates that, compared to actively searching for meaning, the presence of meaning in life functions as a more fundamental and stable protective factor in curbing digital addiction among adolescents. The currently prevalent parenting styles such as “tiger parenting” and “helicopter parenting,” which overemphasize external achievements at the expense of emotional support, may foster a sense of meaninglessness and depressive tendencies in some students (Ye et al., 2024). This, in turn, can drive them to use gaming as a compensatory mechanism to meet their psychological needs. In other words, within the “existential vacuum” created by a lack of PML, the instant feedback from online games—such as achievements, social connections, and virtual identities—becomes a highly attractive “substitute for PML” offering readily accessible psychological fulfillment.

Therefore, this study proposes Hypothesis 2: Presence of meaning in life mediates the relationship between parenting styles and IGD.

### 1.3 Mediating role of self-control

Self-control refers to the active adherence to social norms, delaying immediate impulses, and regulating behavior in unsupervised situations through internal representational thinking and memory recall. It represents a critical transitional stage from external control to mature self-regulation (Kopp, 1982). Self-control functions as a limited psychological resource; individuals possessing greater self-control resources can more effectively inhibit impulses, modify behaviors, or sustain efforts toward long-term goals. Research indicates self-control serves as a protective factor against IGD in adolescents (Kim et al., 2008).

To further understand self-control mechanisms, Hofmann et al. (2009) proposed the Dual-Systems Perspective, offering a crucial framework. This theory posits that self-control reflects the dynamic equilibrium between the impulsive system (pursuing immediate rewards) and the reflective system (considering long-term goals). When self-control is deficient, the impulsive system dominates, leading individuals to prioritize immediate gratification (e.g., immediate gratification from gaming) over long-term benefits (e.g., academic achievement).

Substantial empirical research has demonstrated its core mediating role: self-control serves as a central mediator between parenting styles and addictive behaviors. Existing research indicates that self-control mediates the relationship between parenting styles and IGD (Gui et al., 2025; Zhang et al., 2022; Wang et al., 2024). Self-control is positively associated with presence of meaning in life and negatively associated with search for meaning in life (Li et al., 2019). One study demonstrated that the positive association between self-control and meaning in life was mediated by the perception of having structure in life (Stavrova et al., 2018).

Based on this, Hypothesis 3 proposes that self-control mediates the relationship between parenting styles and IGD.

### 1.4 Testing the chain mediation effect

The whole meaning in life is not only a cognitive experience but also a psychological resource that facilitates behavioral regulation. Research indicates that meaning in life enhances individuals’ adaptability and coping abilities (Haugan, 2014; Krok, 2018; Miao, 2021) and self-regulation capacity (Li et al., 2019; Routledge and FioRito, 2021), particularly significantly promoting self-control (Yu and Du, 2023).

Research indicates that self-control fully mediates the relationship between PML and mobile phone addiction tendencies (Zhang X. G. et al., 2019). If parenting styles overly emphasize external achievements while neglecting emotional support and autonomy development, it may exacerbate students’ lack of PML, trapping individuals in an “existential vacuum (Liu and Wang, 2022; Zhang et al., 2022; Wang et al., 2024).” The instant reinforcement provided by online games—such as achievements, social connections, and virtual identities—becomes an irresistibly attractive “substitute for PML,” offering rapid and easily accessible compensatory psychological satisfaction. Furthermore, research indicates that a lack of meaning in life diminishes self-control (Liu et al., 2022). Individuals lacking a PML find it harder to resist the temptation of gaming’s instant rewards (such as the impulse to “play one more round”), leading to reduced inhibitory control over gaming behavior. Therefore, self-control may function as a “downstream variable,” transmitting the influence of meaning in life toward IGD. Hypothesis 4 proposes that meaning in life and self-control mediate the relationship between parenting styles and IGD in a chain-like manner.

### 1.5 Moderating role of gender

Gender differences are a significant factor in the mechanisms underlying IGD. Gender-related variations play a crucial role in IGD susceptibility, encompassing both biological and psychosocial gender influences. Neuroimaging studies reveal that males exhibit stronger reward system activation (vmPFC, ACC, nucleus accumbens) and weaker impulse control during risk-taking—patterns specific to IGD, while these tendencies are less pronounced in females (Wang et al., 2022).

Psychologically, some males tend to internalize real-life problems internally or cope through online gaming (Zhu et al., 2019). Further research indicates that young males are more susceptible to IGD than females (Bian et al., 2026). This phenomenon is also validated among university students, where male undergraduates exhibit significantly higher rates of IGD than females (Hammood et al., 2025).

Regarding the relationship between addiction and psychological resources, multiple studies have linked “meaning in life” to problematic usage behaviors. On one hand, individuals with higher rates of problematic mobile phone use typically exhibit lower meaning in life (Hu et al., 2022; Zhao et al., 2023). Furthermore, empirical studies among Chinese university students indicate that meaning in life significantly and negatively predicts smartphone addiction, with female students generally scoring higher on meaning in life than males (Ye et al., 2025).

On the other hand, the association between meaning in life and IGD has also been partially validated. For instance, Zhang M. X. et al. (2019) employed a one-year cross-lagged analysis to discover that university students’ meaning in life mediates longitudinally between social support and IGD, while also directly predicting IGD negatively. However, current research predominantly focuses on the relationship between meaning in life and smartphone addiction, with limited exploration of the connection between meaning in life—particularly the PML—and IGD. Additionally, studies indicate differences between genders in the internalization and transformation of psychological resources.

Women typically tend to deeply internalize interpersonal relationships and emotional experiences, forming meaning in life at the cognitive level(PML), which is then transformed into self-control capabilities regulating behavior (Ji et al., 2024; Zhao et al., 2020a,b); whereas males may rely more on external rules or action-oriented strategies (Rose and Rudolph, 2006).

Based on these findings, we hypothesize that the critical point of gender difference may lie in the psychological transformation process of “whether individuals can effectively convert a PML into self-control abilities.” Testing the moderating effect of this pathway can directly validate the theoretical perspective that “women are more adept at converting cognitive psychological resources into behavioral regulation capabilities,” thereby providing a more refined mechanistic explanation for understanding gender differences in IGD risk. Therefore, this study proposes Hypothesis H5: Gender will moderate the chain-mediated pathway through which parenting styles influence IGD via meaning in life and self-control.

In summary, this study examines the relationship mechanisms between parental parenting styles and IGD among college students based on existing research and theories. A thorough understanding of these mediating factors and their role in the relationship between parenting styles and online addiction is crucial, as these insights can significantly contribute to safeguarding the mental health of this population.

This study aims to: Examine the relationship between different parenting styles and IGD among college students. Investigate how a PML and self-control mediate the relationship between parenting methods and IGD, while comparing differences in these mediating effects across parenting styles as well as exploring the moderating role of gender.

### 1.6 Theoretical integration and model positioning

This study integrates the I-PACE model (Brand et al., 2019) as the primary theoretical framework to explain how parenting styles influence Internet Gaming Disorder among college freshmen. The I-PACE model conceptualizes specific Internet-use disorders as the result of dynamic interactions among predisposing person factors, affective and cognitive responses, executive control processes, and reinforcement learning mechanisms. Within this framework, environmental and dispositional factors exert their influence through cognitive and executive pathways that ultimately shape problematic behaviors.

In the present model, parenting styles are positioned as distal environmental factors within the “Person” and contextual layers of the I-PACE framework. Specifically, emotional warmth represents a positive rearing context, whereas rejection and overprotection represent negative rearing environments. These parenting dimensions constitute the antecedent variables in the model.

Presence of Meaning in Life is conceptualized as a cognitive resource and is positioned at the cognitive-response level of the I-PACE framework. Distinct from the motivational dimension of “search for meaning,” PML reflects individuals’ perceived sense of purpose, coherence, and existential significance. In this study, PML serves as the first mediator, representing the cognitive mechanism through which parenting environments shape internal psychological resources.

Self-control is positioned at the executive level of the I-PACE model. Drawing on dual-systems theory, self-control reflects the dynamic balance between impulsive and reflective systems and represents individuals’ capacity to regulate behavior in pursuit of long-term goals. In the proposed sequential pathway, self-control functions as a downstream regulatory mechanism translating cognitive resources into behavioral restraint, with IGD conceptualized as the behavioral outcome.

In addition, gender is specified as a boundary condition within the model. Rather than serving merely as a control variable, gender is hypothesized to moderate the transformation process from cognitive resources (PML) to executive regulation (self-control). In other words, gender may influence the efficiency with which meaning-related cognitive resources are converted into behavioral control capacities.

Furthermore, compensatory Internet use theory complements the I-PACE framework by explaining the functional motivation underlying excessive gaming. When individuals experience psychological deficits or unmet needs in offline contexts, online activities may serve compensatory functions. Within this integrated framework, diminished PML may increase reliance on gaming as a substitute source of purpose or psychological fulfillment.

Taken together, the proposed model delineates a theoretically grounded sequential pathway:

Parenting styles → Presence of Meaning in Life → Self-control → Internet Gaming Disorder, with gender moderating the cognitive-to-executive transformation process. By integrating environmental, cognitive, executive, and behavioral levels within a unified framework, this study provides a more process-oriented explanation of how family environments contribute to IGD risk among emerging adults.

In summary, this study examines the relationship mechanisms between parental parenting styles and IGD among first-year university students based on existing research and theories. A thorough understanding of these mediating factors and their role in the relationship between parenting styles and online addiction is crucial, as these insights can significantly contribute to safeguarding the mental health of this population. Although IGD is a global phenomenon, testing this theoretically grounded model within the Chinese cultural context contributes to evaluating its cross-cultural generalizability.

To address these theoretical and empirical gaps, the present study is guided by the following research questions:

*RQ1*: How do different parenting styles (emotional warmth, rejection, and overprotection) relate to Internet Gaming Disorder among first-year university students?

*RQ2*: Does Presence of Meaning in Life mediate the relationship between parenting styles and IGD?

*RQ3*: Does self-control mediate this relationship, and does it operate as a downstream regulatory mechanism following PML?

*RQ4*: Do PML and self-control jointly form a sequential (chain) mediation pathway linking parenting styles to IGD?

*RQ5*: Does gender moderate the transformation process from cognitive resources (PML) to executive regulation (self-control), thereby influencing the strength of the overall chain mediation?

Based on these research questions and theoretical foundations, we propose the following hypotheses:

*Hypothesis 1*: Positive parenting styles inhibit IGD, while negative parenting styles promote it.

*Hypothesis 2*: Presence of meaning in life mediates the relationship between parenting styles and IGD.

*Hypothesis 3*: self-control mediates the relationship between parenting styles and IGD.

*Hypothesis 4*: meaning in life and self-control mediate the relationship between parenting styles and IGD in a chain-like manner.

*Hypothesis 5*: Gender will moderate the chain-mediated pathway through which parenting styles influence IGD via meaning in life and self-control.

## 2 Subjects and methods

### 2.1 Participants and procedure

The study population consisted of full-time first-year undergraduate students enrolled in standard four-year academic programs. This group represents individuals undergoing the transition from high school to university and has been identified as a high-risk population for Internet Gaming Disorder (IGD) (Gao et al., 2022; Li et al., 2018). To enhance regional representativeness, cluster sampling was employed. Participants were recruited at the class level from two universities in China: one “Double First-Class” university located in western China and one comprehensive undergraduate institution in eastern China.

Data collection was conducted in 2025 using both online and offline methods. In classroom settings, participants were invited to complete the questionnaire by scanning a QR code. Additionally, an online survey link was distributed via the Wenjuanxing platform. Prior to participation, all respondents were informed of the study purpose and were assured of anonymity and voluntary participation.

The inclusion criteria were as follows: (1) full-time first-year undergraduate students enrolled in a standard four-year program; (2) voluntary participation with informed consent; and (3) ability to complete the questionnaire independently. The exclusion criteria included: (1) missing data on key study variables or substantial item omissions; and (2) evidence of careless responding (e.g., extremely short completion times, straight-line responses, or inconsistent answers on reverse-scored items).

A total of approximately 828 questionnaires were distributed. After data screening, 728 valid responses were retained, yielding an effective response rate of 87.9%.

The final sample had a mean age of 18.96 years (SD = 0.82), including 335 males (46.0%) and 393 females (54.0%). Among participants, 34.2% were only children. Urban-origin students accounted for 59.6% of the sample, while 40.4% were from rural areas. Regarding family structure, 91.6% came from two-parent families, 3.3% from father-only households, and 5.1% from mother-only households.

### 2.2 Research instruments

Parenting style: This study employed the Chinese version of the Short-Form Parenting Style Questionnaire (s-EMBU), revised by Jiang et al. (2010). It comprises three dimensions—emotional warmth, rejection, and overprotection—with 21 items in total, including one reverse-scored item (Item 17). Each item presents two distinct parenting approaches (father/mother) for respondents to differentiate and answer. A 4-point scale ranging from “Never” to “Always” was employed. This scale has been widely adopted by Chinese scholars, and it was selected as the research instrument for this study. The overall Cronbach’s *α* for this scale in the study was 0.817. The reliability coefficients were 0.922 for the Rejection dimension, 0.797 for the Overprotection dimension, and 0.946 for the Emotional Warmth dimension. Parenting styles were assessed for validity through KMO sampling adequacy, which was 0.877 (*p* < 0.0001).

Presence of meaning in life: The total meaning in life scale originally developed by Steger et al. (2006), this 10-item scale uses a 1–7 point scoring system. However, the Presence of Meaning subscale is the most widely used measure of this construct (Brandstätter et al., 2012). Given that PML and SML are established as distinct factors (Steger et al., 2006), employing this focused measure allows for a precise investigation into the unique psychological mechanisms of PML, which can inform targeted interventions. Liu and Gan (2010) adapted this scale.

This study employed the Chinese 5-item revised version of the PML Scale, with the item “My life has no definite purpose” reverse-scored. Higher total scale scores indicate greater levels of PML among respondents. The Cronbach’s *α* for this scale in the present study was 0.886. The validity of the PML was assessed through KMO sampling adequacy, which was 0.884 (*p* < 0.001).

Self-control: This study employed the revised Self-Control Scale adapted for Chinese university students by Tan and Guo (2008). They modified Tangney et al.'s (2004) scale to better align with the habits and cultural context of Chinese college students. The revised Self-Control Scale comprises 19 items, with items 1, 5, 11, and 14 scored positively and the remainder negatively. It measures five dimensions: work/study performance, health habits, impulse control, entertainment restraint, and temptation resistance. A 5-point scale was used, ranging from “completely disagree” (1) to “completely agree” (5). This study employs the Self-Control Scale revised by Tan Shuhua et al. The Cronbach’s α for this scale in the present study was 0.931. The validity of self-control was assessed through KMO sampling adequacy, which was 0.95 (*p* < 0.0001).

Internet gaming disorder: This study was assessed using the 9-item internet gaming disorder scale developed by Petry et al. (2014) based on the DSM-5 diagnostic criteria. Item 8 (“using games to escape problems or alleviate negative emotions”) specifically corresponds to the core feature of compensatory use theory. It employs a 5-point scale ranging from 1 (completely disagree) to 5 (completely agree), with a maximum total score of 45. Higher scores indicate greater tendencies toward IGD. In this study, the Cronbach’s α for this scale was 0.914. The validity of the IGD scale was assessed using KMO sampling adequacy, which was 0.877 (*p* < 0.0001).

### 2.3 Data processing

Data were analyzed using IBM SPSS Statistics version 27.0 and Hayes’ PROCESS macro version 4.1. Analyses included common method bias testing, descriptive statistics, Pearson correlations, and both chain and moderated mediation analyses. All statistical tests adopted a significance level of *p* < 0.05. Data cleaning was performed using listwise deletion for cases with missing values on key variables or signs of careless responding (e.g., very short completion times, straight-lining, or inconsistent reverse-scored responses). The final sample included 728 participants (effective response rate = 87.9%). Multicollinearity was checked using variance inflation factors (VIFs) and tolerance values. All VIFs were between 1.05 and 2.11, and tolerance values exceeded 0.47, indicating no multicollinearity issues. Three dimensions of parenting styles—rejection, overprotection, and emotional warmth—were tested in separate models. Mediation and moderated mediation effects were tested using the PROCESS macro (Model 6 and Model 91). Model 6 tested the chain mediation pathway (parenting style → presence of meaning in life → self-control → IGD), while Model 91 tested the moderated mediation effect of gender. Bootstrapping with 5,000 resamples was employed to generate bias-corrected 95% confidence intervals for direct and indirect effects. An effect was considered significant when the confidence interval did not include zero. The moderated mediation effect was evaluated using the index of moderated mediation (Hayes, 2015). Bootstrapping was selected because it does not assume normality of the sampling distribution of indirect effects and is widely recommended for mediation and moderated mediation analyses (Hayes, 2013, 2022, 2015).

### 2.4 Statistical power and sample size

To ensure the robustness of the statistical analyses, an *a priori* sample size estimation was conducted using G*Power 3.1 software (Faul et al., 2009). The estimation was performed separately for the two primary models in the study.

First, for the model testing the chain mediation effect (corresponding to PROCESS Model 6), the analysis was specified as a linear multiple regression (F test). Based on Cohen (1988) conventions, a medium effect size (f^2^ = 0.15) was used as the benchmark. Given an *α* error probability of 0.05, a statistical power (1–β) of 0.95, and a total of 6 predictor variables (including three control variables, one dimension of parenting style, and two mediating variables: presence of meaning in life and self-control), the calculation yielded a minimum required sample size of 146 (Cohen, 1988).

Second, for the moderated chain mediation model (corresponding to PROCESS Model 91), which focuses on testing interaction effects, a more conservative small effect size (f^2^ = 0.02) was adopted for the estimation, as interaction effects are typically smaller. With an α error probability of 0.05, power (1–β) of 0.95, and 6 predictor variables (comprising two control variables, one dimension of parenting style, presence of meaning in life, gender, and the interaction term between presence of meaning in life and gender), the required sample size was calculated to be 652.

With a final valid sample size of 728, our study meets and surpasses the a priori sample size requirements. This number is substantially larger than the 146 needed for the chain mediation model and adequately meets the higher threshold of 652 required to detect the smaller interaction effect in the moderation model. Consequently, the study provides ample power to rigorously test the main, mediating, and moderating effects.

## 3 Results and analysis

### 3.1 Common method variance control and testing

To control for common method variance, this study employed procedures such as anonymous method surveys and reverse scoring. Following data collection, the Harman one-factor test was used to examine common method variance (Zhou and Long, 2004). Analysis revealed that 16 factors had eigenvalues greater than 1, with the first factor explaining 23.909% of the total variance—significantly below the commonly accepted 40% threshold as a benchmark. This indicates that common method bias does not substantially affect the study’s findings, enabling further analysis.

### 3.2 Descriptive statistics and correlation analysis of variables

Descriptive statistics for each variable are presented in Table 1. As shown in Table 1, correlation analysis revealed significant correlations (*p* < 0.01) between parental parenting styles, PML, self-control, and IGD, as detailed in Table 2.

**Table 1:** Correlation matrix (*N* = 728).

Variable	M	SD
1. Gender	1.54	0.499
2. Only child	1.66	0.475
3. Place of origin	1.4	0.491
4. Rejection	8.58	3.18
5. Over protection	16.72	3.88
6. Emotional warmth	20.32	5.45
7. PML	23.45	6.34
8. Self-control	61.53	16.64
9. IGD	19.48	7.42

**Table 2:** Correlation analysis results (*N* = 728).

Variable	1	2	3	4	5	6	7	8	9
1. Gender	1								
2. Only child	0.148**	1							
3. Place of origin	0.047	0.245**	1						
4. Rejection	−0.106**	0.026	0.017	1					
5. Over protection	−0.082*	−0.006	−0.037	0.621**	1				
6. Emotional warmth	−0.008	−0.147**	−0.169**	−0.300**	0.084*	1			
7. PML	−0.047	−0.151**	−0.080*	−0.179**	−0.115**	0.386**	1		
8. Self-control	−0.077*	−0.152**	−0.119**	−0.312**	−0.188**	0.371**	0.593**	1	
9. IGD	−0.130**	0.012	0.038	0.353**	0.238**	−0.231**	−0.282**	−0.515**	1

* Statistical comparisons were made at the 0.05 level (two-tailed) using Pearson’s correlation coefficient. **Statistical comparisons were made at the 0.01 level (two-tailed) using Pearson’s correlation coefficient.

### 3.3 Chain mediation analysis

To test Hypotheses 2–4, a chained mediation model (PROCESS Model 6) with 5,000 bootstrap samples was conducted. Gender, place of origin, and only-child status were included as control variables. All continuous variables were standardized prior to analysis to enhance interpretability. The results are presented in Tables 3, 4.

**Table 3:** The chain mediating effect test.

Predictor variable	PML				Self-control				IGD			
	β	SE	*p*	95%CI	β	SE	*p*	95%CI	β	SE	*p*	95%CI
Gender	0.09	0.07	0.22	[−1.49, 0.34]	−0.13	0.06	0.023	[−3.38, −0.25]	−0.28	0.06	< 0.001	[−2.98, −1.16]
Place of origin	−0.09	0.48	0.8	[−1.50, 0.39]	−0.12	0.06	0.05	[−3.26, −0.03]	−0.01	0.06	0.87	[−1.01, 0.86]
Only child	−0.27	0.08	< 0.01	[−2.72, −0.74]	−0.08	0.06	0.18	[−2.85, 0.55]	−0.09	0.07	0.21	[−1.62, 0.35]
Rejection	−0.18	0.04	< 0.001	[−0.50, −0.22]	−0.22	0.03	< 0.001	[−1.19, −0.70]	0.19	0.03	< 0.001	[0.13, 0.26]
PML					0.54	0.03	< 0.001	[1.04, 1.29]	0.03	0.04	0.44	[−0.05, 0.12]
Self-control									−0.49	0.04	< 0.001	[−0.31, −0.22]
R^2^	0.06				0.41				0.33			
*F*	11.02***				99.28***				58.87***			
Gender	−0.07	0.47	0.35	[−1.36, 0.48]	−0.1	0.06	0.08	[−3.02, 0.19]	−0.31	0.06	< 0.001	[−3.18, −1.35]
Place of origin	−0.1	0.08	0.19	[−1.59, 0.31]	−0.13	0.06	0.03*	[−3.47, −0.16]	−0.01	0.06	0.97	[−0.97, 0.93]
Only child	−0.28	0.08	< 0.001	[−2.80, −0.81]	−0.09	0.6	0.16	[−2.99, 0.49]	−0.08	0.07	0.23	[−1.61, 0.38]
Over Protection	−0.12	0.04	< 0.001	[−0.31, −0.08]	−0.13	0.03	< 0.001	[−0.66, −0.25]	0.13	0.03	< 0.001	[0.07, 0.19]
PML					0.56	0.03	< 0.001	[1.09, 1.34]	0.03	0.04	0.4	[−0.05, 0.13]
Self-control									−0.53	0.04	< 0.001	[−0.33, −0.25]
R^2^	0.04				0.38				0.31			
*F*	7.52***				87.64***				54.57***			
Gender	−0.06	0.07	0.38	[−1.25, 0.47]	−0.09	0.06	0.13	[−2.81, 0.37]	−0.33	0.06	< 0.001	[−3.34, −1.49]
Place of origin	0.01	0.07	0.83	[−0.80, 1.00]	−0.08	0.06	0.19	[−2.78, 0.54]	−0.03	0.07	0.65	[−1.19, 0.74]
Only child	−0.2	0.08	< 0.01	[−2.19, −0.32]	−0.07	0.07	0.28	[−2.69, 0.79]	−0.09	0.07	0.18	[−1.69, 0.32]
Emotional warmth	0.37	0.04	< 0.001	[0.37, 0.54]	0.16	0.03	< 0.001	[0.25, 0.58]	−0.06	0.04	0.1	[−0.13, 0.01]
PML					0.52	0.03	< 0.001	[0.99, 1.26]	0.05	0.04	0.25	[−0.04, 0.14]
Self-control									−0.54	0.04	< 0.001	[−0.34, −0.25]
R^2^	0.16				0.38				0.3			
*F*	34.14***				89.15***				51.27***			

*Statistical comparisons were made at the 0.05 level (two-tailed) using Pearson’s correlation coefficient. **Statistical comparisons were made at the 0.01 level (two-tailed) using Pearson’s correlation coefficient. ***Statistical comparisons were made at the 0.001 level (two-tailed) using Pearson’s correlation coefficient.

**Table 4:** Bootstrap analysis of mediation effects.

Path	Indirect effect size	Effect size	BootstrapSE	95%CI BootCI lower	95%CI BootCI upper
Rejection-PML-IGD	−0.01	1.55%	0.01	−0.02	0.01
Rejection-Self-control-IGD	0.11	31.59%	0.02	0.08	0.15
Rejection-PML-Self-control-IGD	0.05	13.96%	0.01	0.03	0.07
Total indirect effect	0.15	43.97%	0.02	0.11	0.19
Direct effect	0.19	56.03%	0.03	0.13	0.26
Over protection-PML-IGD	−0.004	1.70%	0.01	−0.02	0.07
Over protection-self-control-IGD	0.070	29.83%	0.02	0.03	0.11
Over protection-PML-Self-control-I-GD	0.04	15.61%	0.01	0.01	0.06
Total indirect effect	0.10	43.74%	0.02	0.06	0.14
Direct effect	0.13	56.26%	0.03	0.07	0.19
Emotional warmth-PML-IGD	0.02	7.34%	0.02	−0.02	0.05
Emotional Warmth-Self-control-IGD	−0.09	36.83%	0.02	−0.12	−0.05
Emotional Warmth-PML-Self-control-IGD	−0.11	45.51%	0.02	−0.14	−0.08
Total indirect effect	−0.17	75.00%	0.02	−0.219	−0.13
Direct effect	−0.06	25.00%	0.04	−0.126	0.01

#### 3.3.1 Effects of rejection parenting

Results showed that rejection parenting significantly reduced PML (*β* = −0.18, *p* < 0.001) and directly weakened self-control (*β* = −0.22, *p* < 0.001). In turn, PML positively predicted self-control (*β* = 0.54, *p* < 0.001), and self-control negatively predicted IGD (*β* = −0.49, *p* < 0.001).

Although the direct path from PML to IGD was not significant (*β* = 0.03, *p* > 0.05), rejection parenting still directly predicted IGD (*β* = 0.19, *p* < 0.001).

Bootstrap analyses further indicated that the indirect effect through self-control and the sequential pathway (Rejection → PML → Self-control → IGD) were both significant (see Table 3).

#### 3.3.2 Effects of overprotection parenting

A similar pattern emerged for overprotection. Overprotection significantly negatively predicted PML (*β* = −0.12, *p* < 0.001) and self-control (*β* = −0.13, *p* < 0.001). PML positively predicted self-control (*β* = 0.56, *p* < 0.001), and self-control negatively predicted IGD (*β* = −0.53, *p* < 0.001).

The direct effect of overprotection on IGD remained significant (*β* = 0.13, *p* < 0.001), whereas the direct path from PML to IGD was non-significant.

Bootstrap results confirmed that both the indirect effect via self-control and the chain mediation pathway were significant.

#### 3.3.3 Effects of emotional warmth parenting

Emotional warmth significantly enhanced PML (*β* = 0.37, *p* < 0.001) and directly strengthened self-control (*β* = 0.16, *p* < 0.001). PML further positively predicted self-control (*β* = 0.52, *p* < 0.001), and self-control significantly inhibited IGD (*β* = −0.54, *p* < 0.001). After controlling for the mediators, the direct effect of emotional warmth on IGD was no longer significant (*β* = −0.06, *p* > 0.05), indicating a fully mediated relationship.

Bootstrap analyses showed that the chain mediation pathway (Emotional warmth → PML → Self-control → IGD) was significant (see Table 4). Overall, self-control consistently mediated the relationship between parenting styles and IGD across all three models, supporting Hypothesis 3. The independent mediating role of PML was not significant, failing to support Hypothesis 2. However, the chain mediation pathway from parenting styles to IGD through PML and self-control was significant, supporting Hypothesis 4.

The mediation results indicate (Table 3) that self-control significantly mediates the relationship between the three parenting styles and IGD. However, the mediating effect of PML is not significant, thus H2 is not supported while H3 (the mediating role of self-control) is supported. Furthermore, PML and self-control exert a chain mediation effect on the relationship between parenting styles and IGD. The total indirect effect of the Rejection dimension was 0.15, accounting for 43.97% of the total influence of parenting styles on IGD.

**Figure 1:**
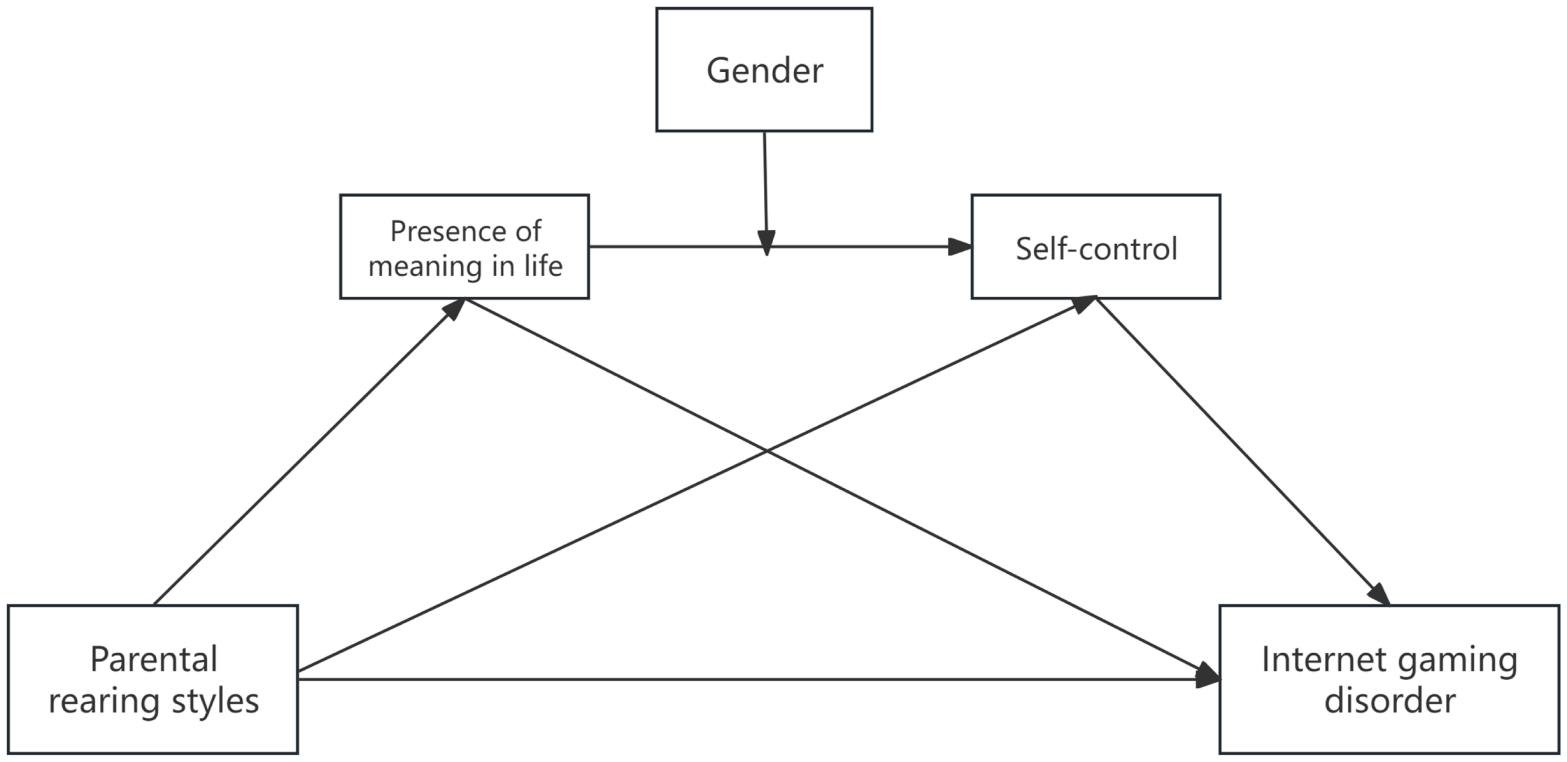
Proposed model.

The total indirect effect of the Overprotection dimension was 0.10, accounting for 43.74% of the total influence of parenting styles on IGD. The total indirect effect of the emotional warmth dimension was −0.17, accounting for 75.00% of the total influence of parenting styles on IGD. Therefore, H4 is supported (Figure 1).

### 3.4 Moderation effect testing

To test Hypothesis 5, a moderated chained mediation model (PROCESS Model 91) with 5,000 bootstrap samples was conducted. Gender was specified as a moderator of the relationship between PML and self-control, while only-child status and place of origin were controlled. Results are presented in Tables 5, 6.

**Table 5:** Moderated chain mediating effect test (*N* = 728).

Predictor variable	PML				Self-control				IGD			
	β	SE	*p*	95%CI	β	SE	*p*	95%CI	β	SE	*p*	95%CI
Only child	−0.29	0.08	< 0.001	[−2.79, −0.84]	−0.08	0.06	0.22	[−2.74, 0.64]	−0.13	0.07	0.06	[−0.26, 0.01]
Place of origin	−0.09	0.08	0.24	[−1.51, 0.38]	−0.12	0.06	0.05	[−3.18, 0.03]	−0.01	0.07	0.86	[−0.14, 0.12]
Rejection	−0.18	0.04	< 0.001	[−0.49, −0.21]	−0.22	0.03	< 0.001	[−1.18, −0.69]	0.21	0.03	< 0.001	[0.15, 0.28]
			
PML					0.3	0.09	0.001	[0.25, 1.04]	0.03	0.04	0.47	[−0.05, 0.10]
Gender					−0.14	0.06	0.001	[−15.70, −3.87]				
Product term					0.16	0.06	0.006	[0.10, 0.58]				
Self-control									−0.48	0.04	< 0.001	[−0.55, −0.40]
R^2^	0.06				0.41				0.31			
F	14.19***				84.73***				64.94***			
Only child	−0.3	0.08	< 0.001	[−2.79, −0.84]	−0.08	0.07	0.19	[−2.74, 0.64]	−0.13	0.07	0.07	[−0.26, 0.001]
Place of origin	−0.1	0.08	0.18	[−1.51, 0.38]	−0.13	0.06	0.04	[−3.18, 0.03]	−0.003	0.07	0.97	[−0.13, 0.13]
Over protection	−0.12	0.04	0.0013	[−0.49, −0.21]	−0.13	0.03	< 0.001	[−1.18, −0.69]	0.14	0.03	< 0.001	[0.08, 0.21]
PML					0.32	0.1	0.001	[0.25, 1.04]	0.03	0.04	0.43	[−0.05, 0.11]
Gender					−0.11	0.06	0.002	[−15.67, −3.55]				
Product term					0.16	0.06	0.006	[0.10, 0 0.60]				
Self-control									−0.52	0.04	< 0.001	[−0.59, −0.44]
R^2^	0.04				0.38				0.29			
F	9.74***				74.96***				58.95***			
Only child	−0.21	0.08	0.006	[−2.79, −0.84]	−0.06	0.07	0.33	[−2.74, 0.64]	−0.14	0.07	0.045	[−0.28, −0.003]
Place of origin	0.02	0.07	0.84	[−1.51, 0.38]	−0.08	0.06	0.21	[−3.18, 0.03]	−0.03	0.07	0.62	−0.17, 0.10
Emotional warmth	0.37	0.04	< 0.001	[−0.49, −0.21]	0.15	0.03	< 0.001	[−1.18, −0.69]	−0.06	0.04	0.07	−0.134, 0.01
PML					0.28	0.1	0.003	[0.25, 1.04]	0.05	0.04	0.26	−0.03, 0.13
Gender					−0.09	0.06	0.003	[−15.173, −3.070]				
product term					0.16	0.06	0.008	[0.088, 0.584]				
Self-control									−0.53	0.04	< 0.001	[−0.61, −0.45]
R^2^	0.16				0.39				0.27			
F	45.27***				76.09***				54.33***			

*Statistical comparisons were made at the 0.05 level (two-tailed) using Pearson’s correlation coefficient. **Statistical comparisons were made at the 0.01 level (two-tailed) using Pearson’s correlation coefficient. ***Statistical comparisons were made at the 0.001 level (two-tailed) using Pearson’s correlation coefficient.

**Table 6:** Moderated chain mediation analysis: conditional indirect effects and index of moderated mediation (*N* = 728).

Parenting style	Male	Female	Index of moderated mediation	95% CI
Rejection	0.09	0.12	0.03	[0.005, 0.064]
Overprotection	0.06	0.07	0.02	[0.003, 0.040]
Emotional warmth	−0.13	−0.17	−0.04	[−0.085, −0.007]

#### 3.4.1 Interaction between PML and gender

Across all three parenting models, the interaction term (PML × Gender) significantly predicted self-control (all ps < 0.01). These results suggest a significant interaction between PML and gender on self-control.

#### 3.4.2 Simple slope analysis

Simple slope analyses showed that PML positively predicted self-control for both males and females (all ps < 0.001). However, the effect was consistently stronger among females across all three parenting models.

**Figure 2:**
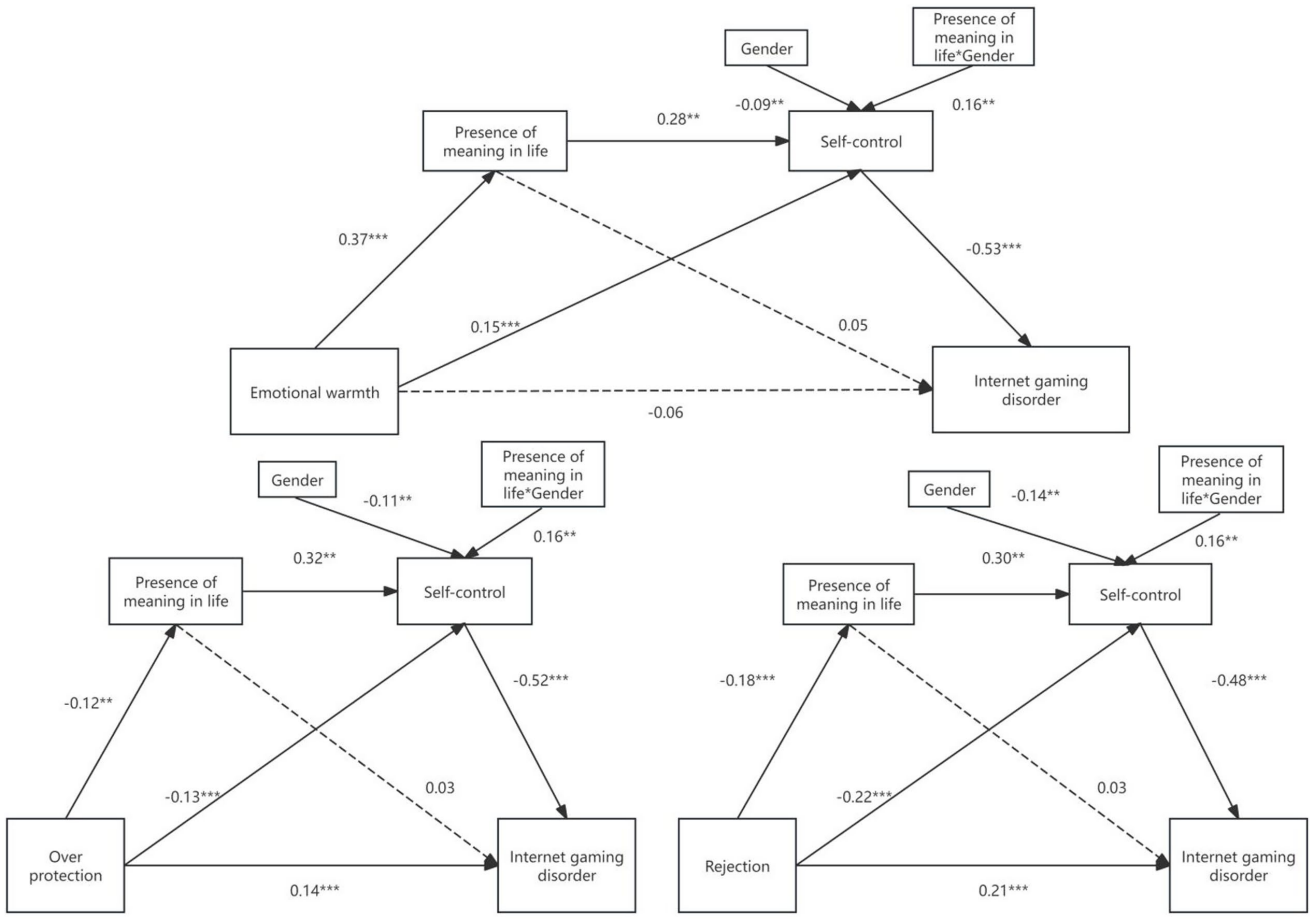
A moderated chain mediating model. **p < 0.01, ***p < 0.001.

**Figure 3:**
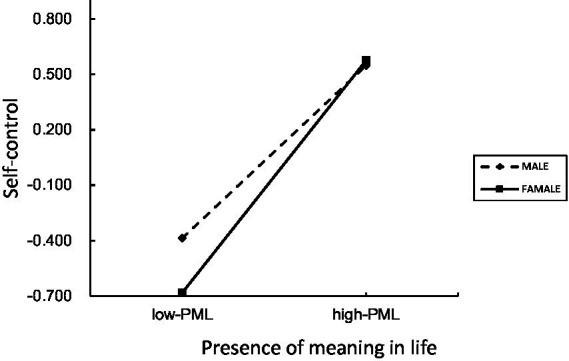
The moderating effect of self-control (rejection).

**Figure 4:**
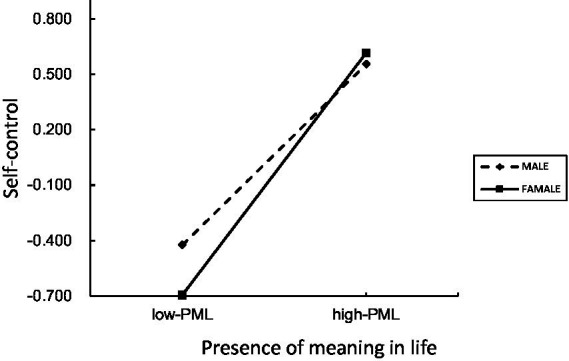
The moderating effect of self-control (over protection).

**Figure 5:**
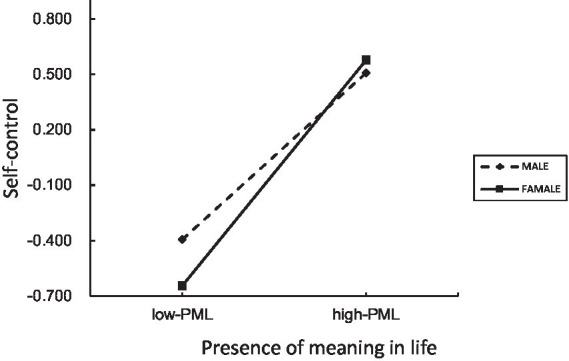
The moderating effect of self-control (emotional warmth).

For example, under rejection parenting, the effect of PML on self-control was stronger for females than for males. Males βsimple = 0.46, t = 10.72, *p* < 0.001, 95% CI [0.37, 0.54]; females βsimple = 0.61, t = 15.45, *p* < 0.001, 95% CI [0.53, 0.69]. A similar pattern emerged for overprotection and emotional warmth (see Figures 2–5).

#### 3.4.3 Moderated chain mediation

As shown in Table 6, this study examined the moderated chain mediation model. To clarify the pattern of moderation, the conditional sequential chain mediation effect was analyzed across gender groups, together with the index of moderated mediation and its 95% confidence interval.

Regarding the emotional warmth dimension: The chain mediation effect was significant for both males (Effect = −0.13) and females (Effect = −0.17). The index of moderated mediation was significantly different between genders (Effect = −0.04, 95% CI [−0.085, −0.007]), indicating a stronger negative (protective) chain mediation effect for females.

Regarding the overprotection dimension: The chain mediation effect was significant for both males (Effect = 0.06) and females (Effect = 0.07). The index of moderated mediation was significantly different between genders (Effect = 0.02, 95% CI [0.003, 0.040]), indicating a stronger risk-inducing chain mediation effect for females.

Regarding parental rejection: The chain mediation effect was significant for both males (Effect = 0.09) and females (Effect = 0.12). The index of moderated mediation was significantly different between genders (Effect = 0.03, 95% CI [0.005, 0.064]), again indicating a stronger risk-inducing chain mediation effect for females.

In summary, the proposed moderated chain mediation model was supported. Gender not only moderates the effect of PML on self-control but also moderates the strength of the chained mediation pathway. Specifically: under positive parental emotional warmth, the protective mediating pathway where females reduce IGD by enhancing PML and self-control is stronger; conversely, under negative parental overprotection and rejecting parenting, the risk-inducing mediating pathway leading to IGD is also stronger among females. Therefore, H5 is supported.

## 4 Discussion

### 4.1 Relationship between parental parenting styles and IGD

Emerging adulthood is a developmental stage characterized by identity exploration, increased instability, and greater vulnerability (Arnett, 2000). Although parental authority typically declines during this phase, parents still exert significant influence through emotional, financial, and interpersonal channels. Recent integrative research on “helicopter parenting” in higher education contexts suggests that over-controlling or over-involved parenting styles may undermine college students’ autonomy and psychological adjustment, increasing the risk of psychological distress and maladaptive behaviors (La Rosa et al., 2025).

Although first-year college students are no longer under direct parental supervision, current findings indicate that early parenting experiences continue to affect the risk of Internet Gaming Disorder (IGD) through internalized psychological processes. Negative parenting styles, such as rejection and overprotection, are significantly positively correlated with IGD, while emotional warmth plays an indirect role through psychological mechanisms. Consistent with previous research on the detrimental effects of over-controlling parenting on the psychological adjustment of emerging adults, this study further suggests that the impact of parenting styles does not diminish upon entering university. Instead, it continues to influence behavioral tendencies through internalized psychological structures. Rather than directly increasing gaming behavior, rejection and overprotection may undermine the development of autonomous self-structure and stable internalized meaning frameworks during emerging adulthood. In the absence of such psychological resources, gaming may function as an accessible compensatory activity that provides structure and immediate reinforcement. However, their effects are primarily indirect, rather than purely direct behavioral effects. This pattern refines compensatory Internet use theory (Kardefelt-Winther, 2014), indicating that compensatory gaming behavior may not only stem from current emotional distress but also from weakened psychological resources in early family environments. In other words, the risk pathway seems to unfold through a decline in internal capabilities rather than through direct parental restrictions or inadequate supervision. These results align with developmental perspectives indicating that parenting practices contribute to the formation of socioemotional competence and self-regulatory capacities that extend beyond adolescence (Baumrind, 1991). Rather than exerting influence through ongoing behavioral monitoring, early family environments may shape relatively enduring psychological orientations that persist into emerging adulthood.

In contrast, once mediating variables were included in the model, emotional warmth could no longer directly predict IGD. This finding suggests that positive parenting styles may play more of a role in fostering protective developmental foundations rather than directly inhibiting maladaptive behaviors. Emotional warmth may cultivate psychological resources that reduce the risk of later addiction, rather than directly suppressing gaming behaviors themselves. This distinction is crucial, as it shifts the focus of the study from behavioral monitoring to the formation of developmental resources.

Our findings suggest that parental influence extends beyond adolescence and continues to shape behavioral vulnerabilities during the transition to adulthood. However, this study does not merely focus on the direct relationship between parenting styles and IGD; it contributes by examining the internal psychological processes through which these influences unfold. Notably, this study was conducted in China, where extended parental involvement and family interdependence are culturally emphasized. Future cross-cultural research should examine whether these mechanisms generalize to other cultural contexts.

### 4.2 Mediating effects of PML and self-control

A key contribution of the present study is to clarify how existential resources and regulatory capacity may jointly account for the association between parenting styles and internet gaming disorder (IGD) among first-year college students. In the correlational analyses, presence of meaning in life (PML) was significantly related to parenting styles and IGD. However, when self-control was included in the full model, the independent indirect effect of PML on IGD was no longer significant. This pattern suggests that PML may be more distal to behavioral outcomes and that its association with IGD may operate primarily through regulatory processes.

PML refers to the extent to which individuals perceive their lives as purposeful, coherent, and significant (Steger, 2012). Compared with situational cognitions, PML can be considered a relatively enduring personal orientation. Within the I-PACE framework, such enduring orientations may be conceptualized as part of the Person (P) component, which includes relatively stable characteristics and belief systems that shape downstream cognitive–affective responses and executive functioning (Brand et al., 2019). From this perspective, PML is more likely to influence problematic gaming indirectly, by shaping the conditions under which individuals engage in self-regulation, rather than directly suppressing gaming behavior.

Importantly, because the present study is cross-sectional, the data do not allow causal or temporal conclusions about the ordering of these processes. Nevertheless, the observed pattern is consistent with a theoretically proposed pathway in which PML is linked to IGD through self-control. This interpretation aligns with dual-system accounts of self-control that distinguish motivational orientation from executive regulation (Hofmann et al., 2009). PML may reflect greater alignment with long-term goals and life direction, but effective restraint in the face of immediately rewarding online activities likely requires sufficient executive control. This view is also consistent with Baumeister’s meaning-based model of self-regulation, which suggests that having a coherent meaning in life provides motivational grounding for long-term goal pursuit, whereas successful inhibition of immediately gratifying behaviors depends on adequate executive control (Baumeister et al., 2007). In this sense, self-control may represent the more proximal mechanism through which existential orientation relates to behavioral outcomes.

Taken together, these findings support a cautious, mechanism-focused interpretation: parenting styles may be associated with IGD through a layered psychological process in which existential orientation (PML) and self-regulatory capacity are jointly involved. This formulation helps reconcile prior work that has often discussed meaning as a protective factor by suggesting that the protective role of PML may be contingent upon its translation into effective self-regulatory processes. Future longitudinal or experimental research is needed to test the temporal ordering implied by this proposed sequence.

### 4.3 Gender differences in the conditional indirect pathway

The present findings indicate that gender moderated the association between presence of meaning in life (PML) and self-control, as well as the conditional indirect effect linking parenting styles to internet gaming disorder (IGD). Specifically, the interaction between PML and self-control was stronger among females than males, resulting in gender differences in the magnitude of the indirect effect. The significant index of moderated mediation further suggests that the indirect association between parenting styles and IGD through PML and self-control varied across gender. Previous research has reported gender differences in emotional processing and coping styles (Ji et al., 2024; Zhao et al., 2020a,b; Root and Denham, 2010), which may be relevant to understanding how psychological resources are mobilized.

### 4.4 Limitation

First, the study adopted a cross-sectional design, which limits the ability to make causal inferences or determine the temporal order of the variables. Although the model structure is based on theoretical assumptions, future research should validate the dynamic developmental relationships between meaning in life, self-control, and Internet Gaming Disorder (IGD) through longitudinal or experimental designs.

Second, all data in this study were collected via self-report questionnaires, which may be subject to social desirability bias or common method bias. Although statistical tests were performed to examine these potential biases, future studies could integrate multi-source data (e.g., parent reports, behavioral measures, or experimental tasks) to enhance the robustness of the findings.

Third, although participants were drawn from two universities in eastern and western China, the sample may not fully represent the diversity of higher education contexts nationwide. Therefore, future research should replicate this study across different cultural backgrounds and educational systems to improve the external validity of the conclusions.

Fourth, potential confounding factors were not fully explored. For example, individual differences in baseline mental health, personality traits, or other environmental influences (such as peer relationships or academic stress) may also contribute to IGD, independent of parenting styles. Future studies could control for these factors to better isolate the unique effects of parenting on IGD.

Finally, while this study examined the moderating role of gender, it did not directly measure potential socialization or developmental variables that may explain gender differences. Future research could incorporate more specific psychological and social mechanisms to deepen our understanding of the sources of gender differences.

## 5 Conclusion

The present study examined the relationships among parenting styles, presence of meaning in life (PML), self-control, and Internet Gaming Disorder (IGD), and constructed a moderated chain mediation model. The findings indicated that negative parenting styles (rejection and overprotection) were not only positively associated with IGD but also indirectly influenced problematic gaming behavior through the sequential pathway of PML and self-control. Emotional warmth, in contrast, exerted its protective role primarily through indirect psychological mechanisms rather than through a direct effect on IGD.

In addition, gender moderated the association between PML and self-control, as well as the overall conditional indirect effect, suggesting that differences may exist in how psychological resources are translated into regulatory capacity across genders.

From a theoretical perspective, this study integrates existential orientation and self-regulatory mechanisms into a framework of psychological adjustment in emerging adulthood, thereby extending current understanding of how parenting styles influence problematic behaviors. The results suggest that the impact of parenting styles on college students’ maladaptive behaviors may not operate as a simple direct effect, but rather unfold through layered psychological processes.

From a practical perspective, the findings highlight the importance of fostering meaning in life and strengthening self-control capacities in efforts to prevent and intervene in IGD among college students. Psychological support strategies in higher education settings may benefit from incorporating gender-sensitive approaches. Enhancing both existential resources and regulatory capacity may contribute to promoting healthier psychological adjustment and developmental outcomes among emerging adults.

## Data Availability

The raw data supporting the conclusions of this article will be made available by the authors, without undue reservation.
